# Chinese doctors’ views on workplace-based assessment: trainee and supervisor perspectives of the mini-CEX

**DOI:** 10.1080/10872981.2020.1869393

**Published:** 2020-12-31

**Authors:** Yuying Liang, Lorraine M. Noble

**Affiliations:** a Department of Medical Education, Affiliated Cancer Hospital and Institute of Guangzhou Medical University, Guangzhou, China; b UCL Medical School, University College London, London, UK

**Keywords:** Mini-CEX, postgraduate medical education, workplace-based assessment, acceptability, China

## Abstract

**Purpose**: This study investigated whether the mini-clinical evaluation exercise (mini-CEX) has been successfully integrated into the Chinese context, following its introduction as part of the national general training programme.

**Materials and methods**: Online questionnaires (N = 91) and interviews (N = 22) were conducted with Year 1 trainee doctors and clinical supervisors at a cancer hospital in China to explore users’ experiences, attitudes and opinions of the mini-CEX.

**Results**” Trainees were more likely than supervisors to report understanding the purpose of the mini-CEX and agree that it encouraged reflection and helped improve overall performance. Both trainees and supervisors felt that it provided a framework for learning, that it was useful in identifying underperformance, and that it informed learning progression. Groups were equally positive about the commitment of their counterpart in the process and valued the focus on detailed feedback. It was perceived as cultivating the learner–teacher relationship. Overall, both groups felt they ‘bought in’ to using the mini-CEX. However, concerns were raised about subjectivity of ratings and lack of benchmarking with expected standards of care.

**Conclusions**: Chinese trainees and supervisors generally perceived the mini-CEX as an acceptable and valuable medical training tool, although both groups suggested enhancements to improve its efficacy.

## Introduction

The nationwide implementation of the Standardised Resident Training Program in 2017 [1], introduced a range of workplace-based assessments (WBAs) into Chinese postgraduate medical education [2]. The ‘mini-CEX’ (mini clinical evaluation exercise) is one such assessment [3,4], involving direct observation of a trainee-patient encounter, with ratings and feedback given by the supervisor across domains such as ‘history-taking’, physical examination, communication, clinical judgement, professionalism and organisation. With strong theoretical validity, the mini-CEX is used internationally [5,6].

When implemented properly, the mini-CEX has positive educational value in clinical training [7,8], but outcomes can be constrained by trainee and supervisor knowledge and attitudes [9]. Acceptance of the mini-CEX differs among users [10,11], but few studies have examined concordance of trainee and supervisor perspectives [12,13].

Furthermore, the acceptability of educational innovation may determine the utility of the mini-CEX in new cultural contexts [14,15]. Studies in southeast Asia have indicated variability in shared understanding and acceptance of the mini-CEX [16,17]. Little is known about how the mini-CEX is implemented in China, as few studies have discussed its validity and reliability [18,19]. As the mini-CEX was designed for use in Western healthcare education, this highlights a need for further investigation into its acceptability and use in practice.

This study therefore aimed to investigate perceptions of the mini-CEX among Chinese trainees and supervisors, particularly considering users’ understanding of this assessment and attitudes towards it.

## Materials and methods

### Participants

All Year 1 trainees and supervisors of Year 1 trainees at a teaching hospital in southeast China were invited to participate. Ninety-one doctors participated: 43/75 (57%) trainees and 48/120 (40%) supervisors; groups were 53% and 63% male, with a median age of 25 years and 52 years, respectively. Participants were based in radiation oncology (38%), surgery (31%), medicine (13%), anaesthetics (12%), gynaecology (3%), radiology (1%) and ultrasound (1%). Supervisors were 26 speciality registrars (years 4–6 following primary medical qualification), 17 consultants (minimum 7 years post-qualification) and 5 chief consultants (minimum 10 years post-qualification). A convenience sub-sample of 22 participants for interviews was recruited: 11 trainees and 11 supervisors; groups were 5/11 and 6/11 male, with a median age of 26 years and 38 years, respectively. Supervisors were six speciality registrars and five consultants (Table 1).

**Table 1.** Participant demographics

	Questionnaire		Interviews	
	Trainees	Supervisors	Trainees	Supervisors
Total number	43	48	11	11
Gender (male)	23	30	5	6
Median Age	25 years	42 years	26 years	38 years
Specialty				
Radiation oncology	18	17	4	3
Surgery	13	15	3	3
Internal medicine	4	8	2	2
Anaesthesiology	7	4	1	2
Gynaecology	1	2	0	0
Radiology	0	1	1	0
Ultrasound	0	1	0	1
Career grade				
Foundation	43	0	11	0
Specialist registrar	0	26	0	6
Consultant	0	17	0	5
Chief consultant	0	5	0	0

### Setting

The host institution was a teaching hospital specialising in the radiological, surgical and medical treatment of adult cancer situated in one of the most developed cities in mainland China. This served a mixed urban and rural population comprising demographically and ethnically diverse communities. Since 2017, The institution introduced WBAs as supplement to the annual exam, which consisted of a knowledge test and Objective Structured Clinical Examination [OSCE). The teaching faculty was therefore offered training of WBAs – including mini-CEX – on an annual basis.

### Measures

A 15-item questionnaire was designed to examine trainees’ and supervisors’ experiences, attitudes and understanding of the mini-CEX, including questions with high reliability adapted from [20,21]. The questionnaire showed good internal consistency, achieving Cronbach’s alpha of 0.95 for both groups [22].

Semi-structured schedules including 9–12 questions for a 30-min interview were devised to explore participants’ perspectives in more depth [9]. Pilot testing of measures was conducted with 12 volunteers (6 trainees, 6 supervisors).

### Procedure

Participant recruitment was conducted through advertisements (via all-staff email, teaching sessions and staff noticeboards) providing a link to the online questionnaire. An invitation to participate in the interviews was included at the end of the questionnaire; volunteers were recruited until the requisite number was reached. Interviews were conducted in a meeting room at the host institution, scheduled as convenient for the participants and audio-recorded with permission.

Qualitative analysis of the interview data was undertaken using principles described by [23]. The interviews were transcribed verbatim by a researcher (YL) to capture the richness of the data set, and read and re-read to identify latent themes reflecting participants’ perceptions, interpretations and attitudes. Initial codes were extracted and free coded in NVivo, before the data were actively searched for themes, and codes grouped into themes by consensus (YL and LN).

### Ethics and data processing

The project was approved by UCL Ethics Committee and the Ethics Committee of Affiliated Cancer Institution and Hospital of Guangzhou Medical University. All participants confirmed informed written consent. Questionnaires were returned anonymously. Audio-recorded interview data were deleted immediately upon transcription. Analysed data contained no identifiable information.

## Results

### Trainee and supervisor experience of the mini-CEX

The majority of trainees (35/43, 81%) indicated a maximum of two previous assessments with the mini-CEX, whilst supervisors had had more experience (Table 2).

**Table 2.** Participants’ experience of the mini-CEX

Number of previous encounters	Questionnaire		Interviews	
	Trainees	Supervisors	Trainees	Supervisors
≤2	35	26	3	3
3–6	3	10	5	6
7–9	4	8	3	1
≥10	1	4	0	1
Total	43	48	11	11

### Trainee and supervisor perceptions of the mini-CEX from the questionnaire

Trainees and supervisors differed in their responses (Mann-Whitney U, z = 3.6, p < 0.001). Trainees were more likely to report a clear understanding of the purpose of the mini-CEX (60% ‘agree’ or ‘strongly agree’) compared to supervisors (48%) (Table 2). On a practical level, trainees were also more likely to report that supervisors initiated the mini-CEX (79% trainees, 48% supervisors) and that there was sufficient time for each assessment (70% trainees, 48% supervisors). The groups showed similar levels of agreement that the patient encounters were directly observed (65% trainees, 67% supervisors) and that feedback was provided after each encounter (70% trainees, 69% supervisors), although supervisors were more likely to report that feedback was immediate (75% supervisors, 65% trainees). The majority of participants in both groups reported that feedback was specific (81% trainees, 75% supervisors) and highlighted weaknesses in performance (74% trainees, 69% supervisors).

Trainees were more likely to report that the mini-CEX encouraged them to reflect (77% trainees, 60% supervisors) and helped to improve their performance (74% trainees, 56% supervisors). The majority of both groups felt that the score reflected the quality of the trainee’s performance (65% trainees, 69% supervisors), that the mini-CEX was useful in identifying underperformance (77% trainees, 70% supervisors), and that it informed learning progression (74% trainees, 69% supervisors). The groups showed similar levels of agreement that their counterpart in the process showed commitment during the assessment (67% trainees, 69% supervisors) and that overall, they ‘bought in’ to the use of the mini-CEX (70% trainees, 69% supervisors).

### Trainee and supervisor perceptions of the mini-CEX from the interviews

Themes related to perceptions of the mini-CEX, its perceived educational impact and attitudes towards its acceptability (Table 4).

**Table 3.** Trainee and supervisor perspectives of the mini-CEX

**Questions**	**Trainees (N = 43)**					**Supervisors (N = 48)**				
	Strongly disagree	Disagree	Neutral	Agree	Strongly agree	Strongly disagree	Disagree	Neutral	Agree	Strongly agree
1. I have a precise understanding of the purpose of the mini-CEX.	0	2	15	20	6	1	7	15	17	6
2. Supervisors initiate the mini-CEX.	0	1	8	21	13	1	6	14	17	6
3. There is sufficient time for each encounter.	1	3	9	21	9	1	6	18	17	6
4. Each encounter is directly observed.	1	4	10	20	8	1	3	12	22	10
5. Feedback is provided after each encounter.	1	3	9	21	9	1	3	11	24	9
6. Feedback is real-time.	1	4	10	20	8	1	6	5	21	15
7. Feedback is content specific.	0	1	7	22	13	1	2	9	26	10
8. Feedback points out weaknesses in performance.	0	1	10	18	14	1	2	12	23	10
9. The mini-CEX helps in prompting reflection.	0	3	7	20	13	1	3	14	21	8
10. Score actually reflects the quality of performance.	1	4	10	20	8	1	3	16	24	9
11. The mini-CEX is useful in identifying under-performance.	0	2	8	22	11	1	4	9	27	7
12. The mini-CEX informs learning progression.	0	4	7	25	7	1	3	11	24	9
13. The mini-CEX helps to improve overall clinical competence.	0	4	7	25	7	0	2	19	22	5
14. The supervisor/trainee shows commitment during mini-CEX encounters.	1	3	10	19	10	1	2	12	21	12
15. In general, mini-CEX gets a buy-in from me.	0	4	9	15	15	0	4	11	19	14

**Table 4.** Themes identified from the trainee and supervisor interviews

Themes	Examples
1. Perceptions of the mini-CEX	
*Interpretation of the purpose*	
Diagnostic tool for learning	‘exemplifying learning strength and weakness’; ‘reflect progress while indicating setbacks’; ‘they watched how you did things and gave feedback, so it is real’
Summary of training in a specific period of time	‘a change in every stage of training’; ‘exemplifies what and how you had learned’; ‘get a real picture of learning’;
Administrative regulation	‘a requirement for both parties’; ‘they [supervisors] are obliged to observe our change in capability now’; ‘now it becomes compulsory and written in paper, they [supervisors] must do that’
A teaching and learning event	‘a standardised learning and teaching activity’; ‘a flexible and authentic learning’; ‘a routine teaching activity’
*Concerns about reliability of scoring*	
Subjective rating	‘lacking a standard for score could undermine the accuracy’; ‘standard varies from trainer’; ‘each tutor holds an individual standard’
Inflated score	‘I don’t intend to hurt someone and that really influences overall score’; ‘they would not fail a trainee by saying the performance is extremely poor’; ‘you want to encourage them, so the overall score could inflate a little’
Calls for training	‘relevant training is definitely necessary’; ‘I guess not all [supervisors] are familiar with the content’; ‘not clear what a standardised encounter is’
*C. Delivery of the mini-CEX*	
Supervisor initiated event	‘supervisor will take care of it [mini-CEX]’; ‘it is my [supervisor’s] responsibility to commence the assessment’; ‘usually they [supervisors] will make plans for that’
Timing of assessment	‘approaching the end of departmental training’; ‘in the middle of rotation’; ‘when I [supervisor] can spare myself from clinical work’
iii. Challenges of allocating time	‘required both of us to try hard to come up with enough time for one encounter’; ‘pressures of service provision make it easier to do it retrospectively’; ‘they [supervisors] are extremely busy’
*D. Observation*	
i. Generating feedback	‘you have to observe to generate feedback’; ‘they [supervisor] watched you doing the job and then fed back’; ‘produce useful tips in feedback’
ii. Effect of corrections on clinical practice	‘you can tell if they [trainees] correct errors in routine work’; ‘I can see [correction] during next performance’
iii. Creates a sense of learning	‘you felt learning when they [supervisors] are watching you’; ‘now you get more attention while they [supervisors] must watch you do things’
*E. Feedback*	
i. Likelihood of provision	‘I get feedback every encounter’; ‘no guarantee you get feedback after each encounter’
ii. Real-time	‘instantly’; ‘immediately’; ‘as soon as it [mini-CEX] is finished’
iii. Honest feedback	‘you must be honest’; ‘it [feedback] is focused on my underperformance’; ‘they [trainees] know that they are here for learning’
iv. Narrative and detail	‘[supervisor] pinpoints some leading language that I used in medical interview’; ‘very detailed and comprehensive’; ‘comments on physical examination or procedures are more specific and explicit’
v. Verification and correction	‘I get corrected right away’; ‘it is normal for them to correct you’; ‘highly appreciate direct feedback’
vi. Overall aim to encourage	‘a slightly larger proportion of positive content [in feedback]’; ‘the main tone of feedback remains positive … they [trainees] need to be encouraged’; ‘balance between positive and constructive’
Educational impact of mini-CEX	
*Framework for assessing and teaching*	‘there is a framework for process or instruction’; ‘broadened my [supervisor’s] interpretation of teaching’; ‘a framework to help us understanding learning task and learning content’
*Guidance for learning*	‘a learning habit that promotes systematic and coherent thinking’; ‘comprehensive illustration of learning tasks and learning content’; ‘flexible and authentic learning: a tool for learning and teaching’
*Provoking reflection for learning and teaching*	‘check what could be amended and needs for special coaching’; ‘leads them [trainees] to think what patients really value’; ‘leads me to tailor my coaching approach to offer best support’
*Co-operation/sharing responsibility*	‘they [trainees] are willing to co-operate’; ‘I am willing to share portions of responsibility in patient treatment with trainees showing good competencies and enthusiasm’; ‘helpful to share treatment responsibility … tutors are more willing to teach, we collaborate better’
*A sense of achievement or self-improvement*	‘you change in every stage of training’; ‘I can see progress in skills because I made a great effort to advance’; ‘I am now confident to illustrate explicitly and consider [assessment] more comprehensively’
*Aligning training with daily work*	‘the most tangible improvement … I can link them to routine work’; ‘compare advice [from supervisor] with practice’; ‘case selected [for assessment] in line with curriculum, usually those common cases with a typical syndrome’
3. Acceptability of mini-CEX	
*Positive*	‘they [trainees] were very engaged in doing it [mini-CEX]’; ‘[Supervisors] are keen to carry out an assessment’; ‘essential for training and I would embrace it’
*Negative*	‘little practical meaning in busy clinical setting and looks like an administrative regulation’; ‘[the supervisor] took the assessment as a tick-box task’; ‘little value in practice’
*Neutral*	‘unclear assessing standard’; ‘may not fit every discipline’; ‘I literally think it is good only if it is infrequent, as it takes a lot of time’

#### Perceptions of the mini-CEX

Participants reported diverse perceptions of the purpose of the mini-CEX, in terms of its various roles as an aid to learning, trainee progression or administrative monitoring. Trainees and supervisors expressed concerns about the reliability of scoring, noting the subjectivity of ratings, and that overly generous scores may be given as a means of encouraging trainees. Interviewees commonly highlighted the need for further training and benchmarking of scores to expected standards.

There was general consensus that initiation of assessment by mini-CEX was the responsibility of the clinical supervisor, although there were diverse views about its timing. This partly reflected supervisors’ views on its formative role, e.g. as a periodic check on progress, or to review the standard of performance attained towards the end of an attachment. However, service pressures also presented challenges to supervisors’ availability.

Almost every interviewee confirmed that trainees were always observed and that observations were used to generate feedback. While supervisors used observation to identify specific areas to improve, trainees felt that being watched helped to create a sense of learning.

Similarly, almost all interviewees agreed that feedback was given after each patient encounter, and all confirmed that feedback was honest. The majority of interviewees reported that feedback was detailed and specific, often providing explicit examples. It was accepted that a key aim of feedback was to correct incorrect practice, in the context of a positive and encouraging approach.

#### Educational impact of mini-CEX

Interviewees consistently described the mini-CEX as a framework for learning and teaching, particularly noting its comprehensiveness and value in ensuring that all domains were covered. Positive benefits were identified in terms of its effects in stimulating reflection, strengthening the learner-teacher relationship, providing a sense of personal achievement, and aligning learning with clinical practice.

#### Acceptability of mini-CEX

No trainees showed negative attitudes towards the assessment, although one reported that some supervisors were ‘sloppy’ in their approach. Two supervisors had misgivings about its structured approach and focus on a single encounter. Generally interviewees reported ‘engaged commitment’ and expressed a willingness to ‘try it more’. Four interviewees expressed a neutral stance, feeling the mini-CEX was generally acceptable, but that some refinement (such as standardisation) was needed.

## Discussion

### Attitudes towards the mini-CEX

The present study found a strong positive attitude towards the mini-CEX among the majority of trainees and supervisors. Trainees valued the mini-CEX more than supervisors, perhaps due to the direct impact on their training, which echoes some earlier studies [20,24,25]. Although some investigators have discussed poor engagement with the mini-CEX [21,26], the majority of participants perceived their counterparts as showing a committed attitude. This may be dependent on who is chosen as the assessor [12,21]. In this context, the clinical supervisor is the default assessor, and the pre-existing relationship may enhance commitment. Traditional Chinese culture values a close bond between the learner and teacher [27]. The present study found that participants viewed organising and leading the mini-CEX as the supervisors’ responsibility, with the trainees’ job to learn and correct mistakes. Such notions create a consensus, resulting in mutual commitment in teaching and learning. Paradoxically, cultures that value doctor autonomy and independence may be less conducive to observation and constructive feedback between members [28,29].

### Mediated tool for teaching and learning in the workplace

The majority of trainees and supervisors perceived the mini-CEX as meaningful, and helpful in avoiding oversights. Teaching and learning in clinical settings are often unstructured due to clinical demands [30]. Any tools that clarify the processes for both learners and teachers are, therefore, essential for learning [31]. The mini-CEX may be particularly helpful for clinical supervisors because the majority are not professional teachers. Its utility in recognizing strengths and underperformance in a number of domains suggests it has value in identifying teaching opportunities across a broad spectrum [32]. The structured approach also helps with assessment, aiding supervisors in recalling their observations and translating their impressions into ratings [33].

The mini-CEX promotes learning through hands-on experience with real-world tasks [25,34]. This is likely to be the greatest advantage perceived by Chinese trainees, as hands-on opportunity is often limited by the strict power distance in social norms as well as the low-trust relationship between Chinese patients and junior doctors [35].

### Making sense of assessment through interaction

This study confirmed previous findings that observing performance allows supervisors to identify learning needs [36] and generate feedback that promotes learning [37]. Supervisors were identified as active information processors and goal directors [33,38], sharing their expectations with trainees following observation [39,40].

Trust is pivotal when analysing performance. Observation offers trainees feedback on the emotional climate of the doctor-patient encounter [39,41]. Trainees also know that supervisors will help if needed. For supervisors, direct observation allows them to ensure patient safety while gaining insight into the trainee’s progress [39].

Both trainees and supervisors valued the element of feedback, which is consistent with evidence that feedback is a powerful tool for learning [42,43]. ‘Task-oriented’ feedback dominated, consistent with previous studies showing that for trainees, content-specific feedback is a ‘shared conversation’, essential to making sense of learning goals and aiding understanding [44].

Most published literature criticises the ‘polite feedback culture’ for withholding honest and constructive feedback in order to ‘save face’ [45]. However, this study indicated that users valued feedback focusing on identifying weaknesses to improve performance. This could reflect cultural norms regarding roles in learning, in which teachers are leaders, guides and masters [27]. This finding also resonates with recent research in East Asian countries that support the educational value of the mini-CEX [15,16]. Traditional teacher-centred Chinese and medical cultures highlight a power differential [15,27,29,46]. Within this context, a supervisor’s expectations and goals represent parts of the curriculum [41] and influence the trainee’s motivation for learning. Consequently, feedback serves as a mediating tool about expectations of acceptable performance [47].

Study participants noted the subjectivity of mini-CEX scoring, which can compromise its utility [17,21]. This highlights a tension between criteria that focus on authenticity and trustworthiness rather than psychometric models that strive to objectify performance and yield reproducible, generalisable judgements [2,10]. Users appeared to operate from differing assumptions of how ‘standardised’ assessments should be [48]. While many trainees felt confident about the mini-CEX rating standard, fewer supervisors felt the same. This divergence may reflect the implicit perception that ratings are summative [2]. This poses a dilemma for supervisors, who also felt a need to encourage trainees at their formative career stage with lenient scoring. It is possible that supervisors held beliefs that were more consistent with traditional principles of scientific measurement [48]. Historically, medicine and science have reflected a positivist paradigm, in which scientific measurement aims to find objective truth [49]. Consequently, it may be unsurprising that supervisors are concerned about the ‘robustness’ of using narrative text for the final judgement [2]. However, the majority of trainees admitted that they were not as concerned about the score because feedback was more direct and useful. Previous studies have not adequately discussed this, possibly because participants believe that judgement in performance assessments such as the mini-CEX is ‘in situ’ and, therefore, can only be understood in context [37]. Trainees may see variations in rating as a source of information about their performance, interpreted as a reflection of supervisors’ experiences [50]. Thus, trustworthiness and authenticity become key players in constructing the meaning and criteria of assessment [2,51]. These are the two most important attributes in Chinese cultural norms for teaching and learning [46], which may mediate users’ concerns with ‘objective ratings’.

### Challenges

The mini-CEX is predicated on the notion that assessment drives learning [8,52]. However, the current study indicated that participants did not fully understand the formative purpose of the mini-CEX, particularly supervisors, a fact that has been acknowledged in numerous studies [8,12,21]. [53], noted that stakeholders’ beliefs in the assessment shape the way they use it. The current study suggests that participants see the mini-CEX as serving mixed purposes. As the majority of mini-CEX evaluations occur towards the end of a rotation, both groups were aware that the assessment outcome could impact the trainee’s progression. Despite these worries, both parties used the mini-CEX as a diagnostic tool and means of identifying underperformance.

The current study identified that time constraints could undermine the perceived educational benefits of the mini-CEX. Trainees may challenge the value of a mini-CEX conducted retrospectively or without direct observation, a repeated finding in global studies of mini-CEX implementation [54].

### Implications and future research

The present study indicated that Chinese participants held positive views about the educational value of the mini-CEX, but there were unresolved concerns about its purpose (formative/summative) and the subjectivity of scoring. Further research could examine how users navigate the potentially competing aims of this process. Future studies may consider other healthcare settings in China [14,55] to examine generalisability of these findings in this new cultural context.

## Conclusion

This study echoed previous research suggesting that formative purpose, direct observation and real-time feedback are crucial components for ensuring effective utilisation of the mini-CEX. Overall, the mini-CEX was welcomed and accepted by Chinese users, although users did not completely understand the notion of formative purpose. The educational value of the mini-CEX appears to be strongly associated with the prevalent cultural norm of learner–teacher relationships based on trust. This study showed that direct observation creates a sense of safety for learning, and moreover, observation is bidirectional as it allows trainees to observe supervisors illustrating corrections.

Some unique cultural attributes about feedback characteristics were revealed, such as preference for mentioning weaknesses and demonstrating correction.

Adequate training for all participants and sufficient time allocation for the mini-CEX were highlighted as implementation needs.

## Practice points

- The utility of mini-CEX is dependent on its acceptability among users and shared understanding of its purpose.
- Overall, the mini-CEX is welcomed and generally accepted in China, with trainees somewhat more positive than supervisors.
- A trustworthy learning-teaching relationship enhanced the educational value of the mini-CEX.
- Faculty training and ensuring time allowance can optimise the full educational potential of the mini-CEX.
